# Development and external validation of a multivariable [^68^Ga]Ga-PSMA-11 PET-based prediction model for lymph node involvement in men with intermediate or high-risk prostate cancer

**DOI:** 10.1007/s00259-023-06278-1

**Published:** 2023-06-01

**Authors:** Urs J. Muehlematter, Lilit Schweiger, Daniela A. Ferraro, Thomas Hermanns, Tobias Maurer, Matthias M. Heck, Niels J. Rupp, Matthias Eiber, Isabel Rauscher, Irene A. Burger

**Affiliations:** 1 Department of Nuclear Medicine, University Hospital Zurich, University of Zurich, Zurich, Switzerland; 2 Institute of Diagnostic and Interventional Radiology, University Hospital Zurich, University of Zurich, Zurich, Switzerland; 3 Department of Nuclear Medicine, Technische Universität München, Klinikum Rechts Der Isar, Munich, Germany; 4 Department of Radiology and Oncology, Faculdade de Medicina FMUSP, Universidade de São Paulo, São Paulo, Brazil; 5 Department of Urology, University Hospital Zurich, University of Zurich, Zurich, Switzerland; 6 Department of Urology, Technische Universität München, Klinikum Rechts Der Isar, Munich, Germany; 7 Department of Urology and Martini-Klinik, Universität Hamburg-Eppendorf, Hamburg, Germany; 8 Department of Pathology and Molecular Pathology, University Hospital Zurich, University of Zurich, Zurich, Switzerland; 9 Department of Nuclear Medicine, Baden Cantonal Hospital, Baden, Switzerland

**Keywords:** Prostate cancer, Lymph node invasion, Prostate-specific membrane antigen positron emission tomography, Prediction

## Abstract

**Purpose:**

To develop and evaluate a lymph node invasion (LNI) prediction model for men staged with [^68^Ga]Ga-PSMA-11 PET.

**Methods:**

A consecutive sample of intermediate to high-risk prostate cancer (PCa) patients undergoing [^68^Ga]Ga-PSMA-11 PET, extended pelvic lymph node dissection (ePLND), and radical prostatectomy (RP) at two tertiary referral centers were retrospectively identified. The training cohort comprised 173 patients (treated between 2013 and 2017), the validation cohort 90 patients (treated between 2016 and 2019). Three models for LNI prediction were developed and evaluated using cross-validation. Optimal risk-threshold was determined during model development. The best performing model was evaluated and compared to available conventional and multiparametric magnetic resonance imaging (mpMRI)-based prediction models using area under the receiver operating characteristic curves (AUC), calibration plots, and decision curve analysis (DCA).

**Results:**

A combined model including prostate-specific antigen, biopsy Gleason grade group, [^68^Ga]Ga Ga-PSMA-11 positive volume of the primary tumor, and the assessment of the [^68^Ga]Ga-PSMA-11 report N-status yielded an AUC of 0.923 (95% CI 0.863–0.984) in the external validation. Using a cutoff of  ≥ 17%, 44 (50%) ePLNDs would be spared and LNI missed in one patient (4.8%). Compared to conventional and MRI-based models, the proposed model showed similar calibration, higher AUC (0.923 (95% CI 0.863–0.984) vs. 0.700 (95% CI 0.548–0.852)—0.824 (95% CI 0.710–0.938)) and higher net benefit at DCA.

**Conclusions:**

Our results indicate that information from [^68^Ga]Ga-PSMA-11 may improve LNI prediction in intermediate to high-risk PCa patients undergoing primary staging especially when combined with clinical parameters. For better LNI prediction, future research should investigate the combination of information from both PSMA PET and mpMRI for LNI prediction in PCa patients before RP.

**Supplementary information:**

The online version contains supplementary material available at 10.1007/s00259-023-06278-1.

## Introduction

Accurate primary staging of prostate cancer (PCa) is important for individualized treatment planning. Current guidelines recommend a bone scan and an abdominopelvic computed tomography (CT) or magnetic resonance imaging (MRI) for non-invasive initial staging [1, 2]. However, novel and potentially more reliable diagnostic procedures are evolving rapidly [3]. [^68^Ga]Gallium-Prostate-specific membrane antigen 11 positron-emission tomography (PET) CT or MRI (further referred to as “PSMA PET” showed a promising diagnostic accuracy for primary staging [4-9].

Despite recent advances in imaging, pelvic lymph node dissection (PLND) during radical prostatectomy (RP) represents the gold standard for nodal staging in PCa. However, the therapeutic and prognostic benefits of extended PLND (ePLND) and PLND still remain controversial [10-13]. PSMA-PET might impact the indication for PLND and its extent but the oncologic benefit is not yet known [14]. The European Association of Urology (EAU) recommended in 2022 ePLND in patients with a risk of lymph node invasion (LNI)  ≥ 7% using the Briganti 2019 nomogram [15]. However, since up to 20% of the patients suffer a complication after PLND, there is a strong need to improve patient selection for PLND [16].

More recently, incorporation of quantitative imaging data from mpMRI or PSMA PET has been proposed to further improve LNI prediction [17-20]. However, there is still only limited data especially on the added value of PSMA PET for LNI prediction.

We aimed to develop and externally evaluate a prediction model using a combination of clinical and qualitative/quantitative information from PSMA PET/CT for prediction of LNI at RP in patients with intermediate to high-risk PCa.

## Material and methods

### Study design

This is a retrospective, dual-center study reported according to the current guidelines [21]. We developed three LNI prediction models in a training cohort, selected the best performing model (including a clinically meaningful risk threshold), and applied it to a validation cohort. The performance of the model was compared to the performance of available LNI prediction models [17, 18, 22-25-]

### Source of data and study population

Patient data from two tertiary referral centers served as data source for the training cohort (Klinikum Rechts der Isar, Technical University of Munich, Munich, Germany) and the validation cohort (University Hospital Zurich, University of Zurich, Zurich, Switzerland).

For the training cohort, the retrospective analysis was approved by the Ethics Committee of the Technical University Munich (permit 5665/13). For the validation cohort, all patients gave a general written informed consent for retrospective use of their data (Ethics Commission of the Canton of Zurich, Switzerland, BASEC Nr. 2018-01284).

We used pre-existing cohorts at both centers that were collected for works on T- and N staging in PCa regarding the training cohort and patient selection for ePLND in the validation cohort, respectively. We extended these cohorts with consecutive new patients. In both cohorts, consecutive PCa patients with histologically proven (D’Amico criteria) intermediate or high-risk PCa who underwent PSMA PET for primary staging followed by RP and ePLND were retrospectively identified (training cohort *n* = 192 between January 2013 and June 2017, validation cohort *n* = 96 between April 2016 and July 2019). Patients with missing biopsy data (training cohort *n* = 19) and without written consent for retrospective use of their data (validation cohort *n* = 6) were excluded, leading to a final training cohort of 173 patients and validation cohort of 90 patients. Ninety-four of 173 patients of the training cohort were part of published works on T- and N staging in PCa patients [6, 8]. Sixty of 90 patients of the validation cohort were part of a published work on patient selection for ePLND in PCa [19].

In the training cohort, all included patients underwent mpMRI-targeted /standard 12-core biopsy followed by PSMA PET and RP with ePLND. ePLND was performed according to a predefined template including bilateral, separate dissection of the obturator fossa, external iliac, internal iliac, and common iliac vessels with the femoral canal and the aortic bifurcation as proximal and distal limits, respectively.

In the validation cohort, all patients with intermediate and high-risk PCa underwent a combined mpMRI-targeted/saturation biopsy (min. 40 cores) followed by PSMA PET and RP with ePLND. ePLND was performed as previously reported [26].

Because of the different biopsy approaches, difference in pathological upgrading at RP between the two cohorts was assessed.

In both cohorts, patients underwent PSMA PET according to standard procedure guidelines and no therapy has been initiated between PET and RP [8, 19].

### Outcome

The predicted outcome was LNI at RP with ePLND. In the training cohort, RP was performed mainly open or robotic (< 10%) with ePLND to a template of 8 predefined anatomical fields. For the validation cohort, all surgical procedures were performed via robot-assisted transperitoneal laparoscopic RP with ePLND as described earlier [26]. In both cohorts, the removed LN were assessed for LNI by specialized uropathologists.

### Predictors

The following data was collected for each patient: Clinical parameters: age [years], PSA value at the time of PSMA PET [µg/l], highest WHO/International Society of Urological Pathology (WHO/ISUP) grade group (grade groups 1–5) [27] at systematic/targeted biopsy; Quantitative ^68^Ga-PSMA-11-PET parameters of the primary tumor of the prostate: maximum standard uptake value (SUV_max_), volume-based PSMA PET parameters were assessed using an absolute cut-off at SUV ≥ 4, yielding PSMA positive volume (PSMA_vol_, [cm^3^]), and total PSMA accumulation (PSMA_total_ = PSMA_vol_ x SUV_mean_) as described earlier [19]. Qualitative PET information: the conclusion of the interpreting physician regarding LNI, i.e., PSMA PET report N-status (0, LNI negative; 0.5, equivocal for LNI; 1, LNI positive, unitless) according to Fanti et al. [28]. Since PET reporting was not standardized during the inclusion time, all PSMA PET at both centers were reassessed by two nuclear medicine physicians in consensus and blinded regarding the outcome.

### Data for comparison with published models

Additional data was extracted to compare the model’s performance with published models in the validation cohort as listed in Supplemental Table S1.

### Missing data

Cases with missing data were omitted (i.e., complete-case analysis).

### Model development and selection

We developed three models for LNI prediction. In the first model, we combined all clinical and quantitative PET information (Model_Clinical_PET). In the second model, we added the PSMA PET report N-status as an additional variable to the first model (Model_Clinical_PET_Report). In the third model, we added the PSMA PET report N-status to the first model as a combined (ensemble) model (Model_Clinical_PET/Report) [29]. WHO/ISUP grade groups (i.e., grade groups 1–5) and the PSMA PET report N-status were treated as continuous predictor. In an internal validation, we assessed the model’s discrimination ability. We chose the model with the highest internal discrimination ability for external validation. A probability threshold for clinical application was estimated from the training cohort and applied to the validation cohort.

### External validation

In the validation cohort, we assessed the model’s performance in terms of model calibration, model discrimination ability, and clinical application.

### Model comparison with published prediction models

In the validation cohort, the selected PSMA PET model was compared with six prediction models in clinical use (mpMRI-based models, 2019 Briganti nomogram [17], Draulans et al. nomogram [18]; conventional models, MSKCC Pre-Radical Prostatectomy nomogram [22], the updated Partin tables (v.2016) [23], the Roach formula [24], and the Winter nomogram [25]). For the probability of LNI of the MSKCC Pre-Radical Prostatectomy nomogram, we used the model properties published online (https://www.mskcc.org/nomograms/prostate/pre_op/coefficients, Model N 6599/11816, updated 01/2020). For all other prediction models, the probability for LNI was calculated using the published model formulas. The final selected model was compared to the published prediction models regarding calibration, discrimination, and clinical application.

### Statistical analysis

The patient’s characteristics were summarized using the mean, median, standard deviation, and interquartile range (IQR), as appropriate. Comparison of patients’ characteristics was conducted by a two-sample *t*-test or Mann-Whitney *U* Test for continuous variables and χ^2^ test for categorical variables.

Predictors were investigated for linearity/multicollinearity using scatter plots/generalized-variance-inflation calculations, respectively. We used a multivariable logistic regression model for the Model_Clinical_PET and Model_Clinical_PET_Report model, and two separate logistic regression models for the ensemble model (Model_Clinical_PET/Report) that were averaged using weights that were optimized using nonlinear optimization [29, 30]. Model calibration was assessed at mean, weak, and moderate level including the Brier score and Spiegelhalter’s z [31]. Discrimination ability was assessed using AUC and clinical application using decision curve analysis, (DCA) respectively. Combined model calibration and discrimination was assessed using the index of prediction accuracy (IPA) [32]. AUC were compared using the Delong method [33]. For the internal validation, we applied a 10-times repeated tenfold cross-validation.

The probability threshold for the final model was selected using a 10-times repeated tenfold cross-validated DCA in the training cohort and by trying to match the reported spared ePLND (65.5%) and missed LNI (12.2%) for the 5% threshold for the 2012 Briganti model development [34].

For the external validation, the final model was trained on the training cohort and was applied to the validation cohort.

A 2-tailed *P* value of  < 0.05 was used to determine the statistical significance. We performed all statistical analysis in R version 4.0.5 (R Core Team (2021) R: A language and environment for statistical computing, Vienna, Austria).

## Results

### Patients’ characteristics and qualitative PSMA-11 PET performance

**Fig. 1:**
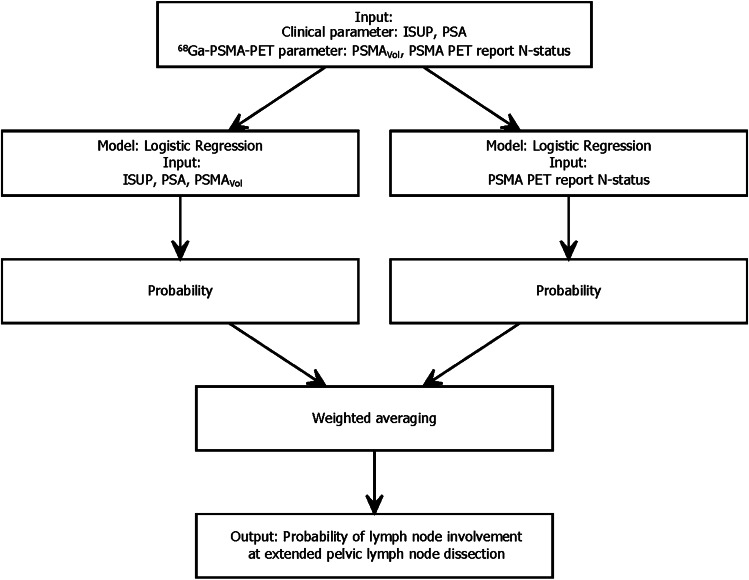
Flow-chart of the ensemble (combined) model (Model_Clinical_PET/Report). The input (predictors) is processed within two separate logistic regression models and combined using averaged weights that were optimized using nonlinear optimization

The data assembly process is demonstrated in Fig. 1. A total of 173 patients were available for the training cohort and 90 patients for the validation cohort. Table 1 lists all patient’s characteristics. The patients’ age was significantly higher in the training cohort (mean age 71.2 vs. 64.7 years, t = -7.34, df = 212.84, *P* < 0.001). Furthermore, the biopsy WHO/ISUP grade group distribution differed between the two cohorts with more ISUP grade 1 biopsies in the training group (ISUP grade 1, 8 vs. 0%, χ2 = 11.196, *P* = 0.02). There was no significant difference regarding pathological upgrading after RP (17 vs. 16%, χ2 = 0.0376, *P* = 0.85). The number of removed lymph nodes during ePLND did not differ between the training and validation cohort (mean 24.1 vs. 23.7, *P* = 0.76).

**Table 1:** Patient characteristics

Characteristics		Training cohort (*n* = 173)			Validation cohort (*n* = 90)			*P*
		LNI-	LNI +	P	LNI-	LNI +	P	
Age	Mean	72	74	0.20	65	63.7	0.46	** < 0.001**
	Range	50 – 85	53—89		51 – 79	50 – 76		
	SD	7.9	7.2		6.02	7.30		
PSA	Median	10	14.7	**0.02**	8.5	14.0	**0.03**	0.15
	Range	1.82—93.9	0.57 -100		1.22 – 55.0	2.08 – 143		
	IQR	7.92	18.87		7.9	13.5		
Highest biopsy ISUP^a^	1	12	2	0.15	-	-	**0.01**	**0.02**
	2	24	9		9	5		
	3	23	11		18	2		
	4	35	14		32	5		
	5	23	20		10	9		
cT	T1c	-	-	-	53	9	**0.01**	-
	T2a	-	-		15	11		
	T3a	-	-		1	1		
SUV_max_	Median	10.7	12.73	0.08	10.2	18.0	** < 0.001**	0.92
	Range	0—122.8	4.1- 42.6		0 – 45.7	6.2 – 48.4		
	IQR	11.3	11.6		10.6	14.6		
PSMA_vol_	Median	3.5	8.4	** < 0.001**	3.0	12.4	** < 0.001**	0.97
	Range	0—89.5	0.04—64.5		0 – 20.4	0.61 – 39.5		
	IQR	5.2	24.8		5.2	19.0		
PSMA_total_	Median	21.2	49.3	** < 0.001**	13	89.9	** < 0.001**	0.51
	Range	0.0—863.8	0.2—643.3		0 – 108.6	3.0 – 412.1		
	IQR	37.5	166.1		44.5	148.6		
PSMA PET report N-status	LNI negative	116	17	** < 0.001**	64	10	** < 0.001**	0.49
	Equivocal	0	9		3	2		
	LNI positive	1	30		2	9		
LNI	0	117	0	-	69	0	-	0.17
	1	0	56		0	21		

^a^At targeted/systematic biopsy

Values in bold indicate statistical significance

Eighteen patients of the validation cohort had missing data concerning the 2019 Briganti model and 4 patients for the Draulans et al. model. Patient characteristics of the complete case cohorts of these models are demonstrated in the Supplementary Table S2.

Supplementary Table S3 lists the qualitative PSMA PET results.

### Model development and selection

Age and SUV_max_ showed a non-linear relationship with the logit of the outcome and PSMA_total_ showed the highest collinearity. Therefore, we excluded these predictors. The AUC for predicting LNI was consistently high with all three models during internal validation (Model_Clinical_PET 0.721 (CI 0.694-0.747) (ISUP, PSA, PSMA_vol_ as predictors), Model_Clinical_PET_Report 0.816 (CI 0.791-0.841) (ISUP, PSA, PSMA_vol_, PSMA PET report N-status as predictors), and Model_Clinical_PET/Report 0.842 (CI 0.82–0.865) (ISUP, PSA, PSMAvol, PSMA PET report N-status as predictors, combined in two models). Supplemental Table S4 lists the full model specifications. Model_Clinical_PET/Report (Fig. 1, Supplemental Table S4) showed the highest internally validated AUC (0.842 CI 0.82–0.865) and was selected for further analysis. The internally cross-validated DCA of this model showed a better net benefit (NB) than either the treatment or no treatment schemes when the threshold probability was  ≥ 0.15 (Supplementary Fig. 1). A threshold probability of  ≥ 17% with an estimated spared ePLND of 54.3% and missed LNI of 19.1% fitted best the reported corresponding values of the 5% threshold of the 2012 Briganti model (spared ePLND of 65.5% and missed LNI of 12.2%) and was chosen as threshold for external validation.

### External validation

**Fig. 2:**
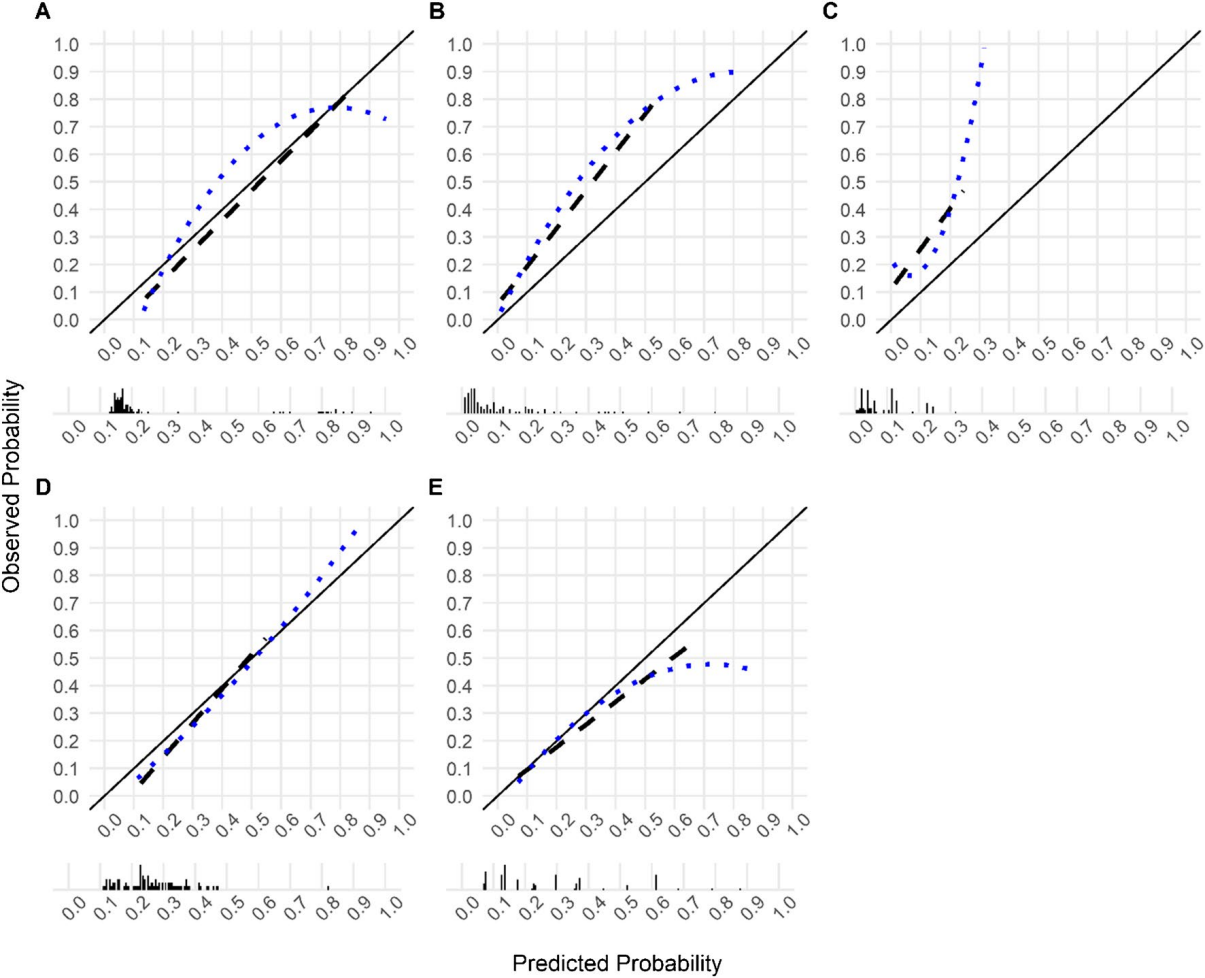
Model calibration plots of predicted probability versus observed probability of lymph node involvement for (**A**) the proposed model (Model_Clinical_PET/Report), (**B**) the MSKCC Pre-Radical Prostatectomy nomogram, (**C**) the updated Partin tables (v.2016), (**D**) the Roach formula, and (**E**) the Winter nomogram. The dotted lines represent the LOESS fit, the dashed lines represent a straight fit. The black bars denote the distribution of predicted probabilities

In the external validation, the ensemble model Model_Clinical_PET/Report showed good calibration-in-the-large (event rate = 0.30, average predicted risk = 0.28) and there was no evidence of systematic over- or underfitting (Intercept = -0.297, Slope = 1.095, *p* = 0.41; Brier score 0.12, Spiegelhalter’s z -0.89). However, calibration curve showed an overestimation of the risk of LNI among patients with observed LNI probability below 0.22 and underestimated the risk of LNI among patients with observed LNI probability above 0.22 (Fig. 2). The model showed a high discrimination ability for LNI (AUC 0.923, 95% CI 0.863–0.984) (Table 2).

**Table 2:** Area under the curve of the proposed model and other lymph node invasion prediction models in the validation cohort

N	Model name	AUC (95% CI)
90	MSKCC	0.824 (0.710–0.938)
	Partin v.2016	0.644 (0.505–0.783)
	Roach	0.700 (0.548–0.852)
	Winter	0.720 (0.590–0.851)
	Model_Clinical_PET/Report	0.923 (0.863–0.984)
67	2019 Briganti	0.786 (0.607–0.966)
	Model_Clinical_PET/Report	0.900 (0.815–0.986)
86	Draulans et al	0.822 (0.700–0.944)
	Model_Clinical_PET/Report	0.921 (0.859–0.983)

Table 3 lists the results of the model application to the validation cohort according to thresholds between a predicted probability of LNI of 0 and 0.30. By using the previously estimated cut-off of  ≥ 0.17, 45 ePLNDs (45/90, 50%) would have been avoided, 44 of them in patients without LNI (44/69, 63.8%) and one in a patient with LNI (1/21, 4.8%), respectively.

**Table 3:** Analyses of the proposed model-derived (Model_Clinical_PET/Report) cut-offs used to discriminate between patients with or without lymph node involvement confirmed at extended pelvic lymph node dissection

Calculated probability of LNI % (cut-off)	Number of patients, n (%)						Sensitivity	Specificity	Net benefit
	Below the cut-off (ePLND not recommended)			Equal to or above the cut-off (ePLND recommended)					
	Total	Without LNI	With LNI	Total	Without LNI	With LNI			
0–13	0 (0)	0 (0)	0 (0)	90 (100)	69 (100)	21 (100)	1.000	0.000	0.119
14	4 (4.4)	4 (5.8)	0 (0)	86 (95.6)	65 (94.2)	21 (100)	1.000	0.058	0.116
15	15 (16.7)	15 (21.7)	0 (0)	75 (83.3)	54 (78.3)	21 (100)	1.000	0.217	0.127
16	28 (31.1)	28 (40.6)	0 (0)	62 (68.9)	41 (59.4)	21 (100)	1.000	0.406	0.147
**17**	**45 (50)**	**44 (63.8)**	**1 (4.8)**	**45 (50)**	**25 (36.2)**	**20 (95.2)**	**0.952**	**0.638**	**0.165**
18	54 (60)	53 (76.8)	1 (4.8)	36 (40)	16 (23.2)	20 (95.2)	0.952	0.768	0.183
19	61 (67.8)	59 (85.5)	2 (9.5)	29 (32.2)	10 (14.5)	19 (90.5)	0.905	0.855	0.185
20	65 (72.2)	62 (89.9)	3 (14.3)	25 (27.8)	7 (10.1)	18 (85.7)	0.857	0.899	0.181
21	68 (75.6)	63 (91.3)	5 (23.8)	22 (24.4)	6 (8.7)	16 (76.2)	0.762	0.913	0.160
22	69 (76.7)	63 (91.3)	6 (28.6)	21 (23.3)	6 (8.7)	15 (71.4)	0.714	0.913	0.148
23	71 (78.9)	63 (91.3)	8 (38.1)	19 (21.1)	6 (8.7)	13 (61.9)	0.619	0.913	0.125
24	72 (80)	64 (92.8)	8 (38.1)	18 (20)	5 (7.2)	13 (61.9)	0.619	0.928	0.127
25	72 (80)	64 (92.8)	8 (38.1)	18 (20)	5 (7.2)	13 (61.9)	0.619	0.928	0.126
26	73 (81.1)	64 (92.8)	9 (42.9)	17 (18.9)	5 (7.2)	12 (57.1)	0.571	0.928	0.114
27	73 (81.1)	64 (92.8)	9 (42.9)	17 (18.9)	5 (7.2)	12 (57.1)	0.571	0.928	0.113
28	73 (81.1)	64 (92.8)	9 (42.9)	17 (18.9)	5 (7.2)	12 (57.1)	0.571	0.928	0.112
29	73 (81.1)	64 (92.8)	9 (42.9)	17 (18.9)	5 (7.2)	12 (57.1)	0.571	0.928	0.111
30	73 (81.1)	64 (92.8)	9 (42.9)	17 (18.9)	5 (7.2)	12 (57.1)	0.571	0.928	0.110

*LNI*, lymph node involvement; *ePLND*, extended pelvic lymph node dissection

Values in bold indicate the proposed threshold

### Model comparison with published prediction models

The proposed model showed similar calibration compared to the conventional and mpMRI-based models. Calibration curves/calibration characteristics are depicted in Fig. 2A-E/Supplementary Table S3 and Supplementary Fig. 2A-D/Supplementary Table S5/6 for comparison with conventional and mpMRI-based LNI prediction models, respectively.

The proposed model showed significantly higher discrimination (AUC 0.923, 95% CI 0.863–0.984) compared to all conventional prediction models except the MSKCC model (AUC 0.824, 95% CI 0.710–0.938), and non-significant higher AUC compared to the mpMRI-based LNI prediction models (Table 2).

The proposed model showed higher combined discrimination and calibration (IPA 0.35) compared to the conventional models and a combined discrimination and calibration higher than the Draulans et al. model (IPA 0.31) and lower than the Briganti 2019 model (IPA 0.37). All IPA values are depicted in Table S5/6.

**Fig. 3:**
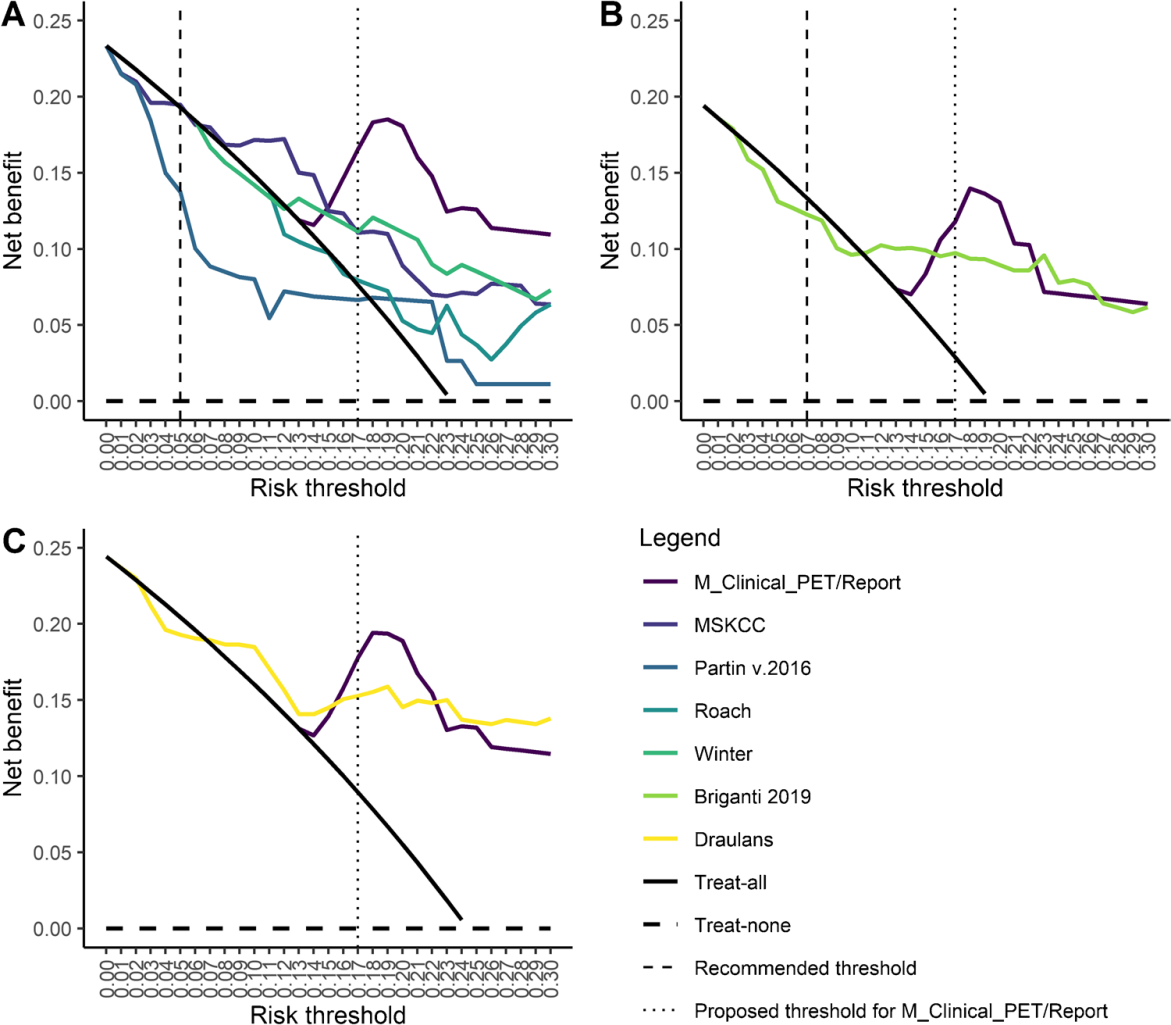
Decision curve analysis (DCA) of the proposed model (M_clinical_PET/Report) compared to (**A**) conventional nomograms, (**B**) the 2019 Briganti nomogram, and (**C**) the Draulans et al. nomogram. The DCA depicts the net benefit (NB, *y*-axis) of a model or a strategy (treat-all or treat-none with ePLND) according to a risk threshold (*x*-axis). Of the conventional models (**A**), only the MSKCC model showed a higher NB compared to the treat-all strategy at the recommended threshold of 5%. However, at this threshold, the net benefit of the MSKCC nomogram (0.195) was only slightly above the treat-all strategy (0.193), meaning that one can perform 195-192 = 3 more beneficial ePLND (out of 1000 patients) when using the MSKCC nomogram). At a threshold of  ≥ 17%, the net benefit was 0.165 for the proposed model and 0.076 for the treat-all strategy, meaning that one can perform 165-76 = 89 more beneficial ePLND (out of 1000 patients) when using the proposed model. Of the mpMRI-based models (**B**, **C**), the 2019 Briganti nomogram (**B**) showed a lower net benefit compared to the treat-all strategy at the recommended threshold of 7% and the Draulans et al. nomogram (**C**, no recommended threshold available) showed a consistently better net benefit (0.189) compared to the treat-all strategy at a threshold of 7% and above

DCA revealed a high NB (0.165) of the proposed model compared to the treat-all strategy at the proposed threshold of  ≥ 17% (Fig. 3). Of the conventional models, only the MSKCC Pre-Radical Prostatectomy nomogram showed higher NB compared to the treat-all strategy at the recommended threshold of 5% (Fig. 3A). Of the mpMRI-based models, the 2019 Briganti nomogram showed a lower NB compared to the treat-all strategy at the recommended threshold of 7% and the Draulans et al. nomogram (no recommended threshold available) showed a consistently better NB compared to the treat-all strategy at a threshold of 7% (NB 0.189) and above (Fig. 3B, C).

## Discussion

In this study, we developed and externally evaluated a multivariable prediction model including quantitative and qualitative information from PSMA PET for predicting LNI at RP with ePLND as reference and assessed its performance against conventional and mpMRI-based prediction models. Our results demonstrated that a model including imaging parameter from PSMA PET might improve models that are solely based clinical parameters. This is consistent with previous reports that assessed the inclusion of mpMRI parameters into prediction models for LNI [17, 18]. Our study introduces an innovative approach to predict LNI by combining PSMA PET reporting by a nuclear medicine physician with readily available quantitative PSMA PET parameters and clinical parameters. A web-calculator to determine the LNI probability according to the proposed model is available under https://psma-pet.com/predict (this calculator should only be used for research purposes).

Our ensemble model incorporating PSA, highest biopsy ISUP, PSMA_Vol_ and the PSMA PET report N-status yielded high sensitivity (0.95) and moderate specificity (0.64) for LNI detection at the proposed threshold of  ≥ 0.17. These results are comparable with the external validation of the 2019 Briganti nomogram (sensitivity of 0.97 and specificity of 0.61) [35]. Our results suggest that especially incorporating the LNI status of the imaging report may improve prediction models, which is in line with previous reports regarding esophageal cancer [36]. We suppose that a combination of predictors from both, mpMRI and PSMA PET might be of value in LNI prediction.

The proposed threshold (≥ 17%) appears rather high compared to recommended cut-offs of 5% for conventional nomograms and 7% for the 2019 Briganti nomogram. However, the external validation shows that this threshold led to reliable results (50% of ePLND spared, with a risk of missing only 4.8% LNI) despite very probable differences between the training and validation cohort (e.g., calibration of the PET scanner). Moreover, a threshold of  ≥ 19% in the validation cohort would have yielded an even higher NB (0.185) and would have spared more ePLND (67.8%), with a risk of missing 9.5 LNI. However, the optimal recommended threshold should also be based on clinical reason, and it is questionable if a potentially higher number of missed LNI is clinically acceptable. Therefore, we suppose that the model’s calibration and the threshold should be further investigated in a larger external cohort.

The potential of imaging variables for predicting LNI in PCa has been reported almost 20 years ago using neural networks [37]. Recently, advanced machine learning algorithms have been reported for LNI prediction in PCa [38, 39]. Cysouw et al. reported an internally validated AUC of 0.86 for LNI in intermediate- to high-risk PCa using PSMA PET radiomics [38]. We think that the sophisticated application of more complex models hinders its transition to clinical practice.

Our study has several limitations. Cases for which ePLND was not performed were not included, causing a selection bias. However, all LNI prediction models, which we used for comparison, are also based on PLND. Our proposed model is based on [^68^Ga]Ga-PSMA-11 PET, which is costly, not yet a standard procedure at many institutions and must be interpreted with care to avoid false positive findings. However, it may be included into widescale practice soon. Moreover, the proposed model is based on [^68^Ga]Ga-PSMA-11 and we did not assess its performance with other PSMA tracers. Moreover, we did not investigate potential bias introduced by different PET scanner and both, the training and the validation cohort were of relatively small sample size, which might have led to bias and may limit the generalizability of our results. The two cohorts differentiated regarding preoperative biopsy (mpMRI-targeted with saturation biopsy versus mpMRI-targeted / standard 12-core biopsy) and surgical approach for RP; however, we did not find a significant difference regarding pathological upgrading after RP, number of removed lymph nodes or LNI rate, respectively. Lastly, because of missing data, we could not directly compare the two mpMRI-based models.

Our results indicate that combining clinical and qualitative/quantitative ^68^Ga-PSMA-11 information may improve LNI prediction in intermediate to high-risk PCa patients undergoing primary staging. The proposed model with a  ≥ 17% threshold yielded a good performance compared to conventional and mpMRI-based models, sparing half of all ePLNDs with a risk of missing only  < 5% LNI. Future research should investigate the combination of information from both PSMA PET and mpMRI for LNI prediction in larger patient cohorts with PCa before RP.

## Supplementary Information

Below is the link to the electronic supplementary material.

Supplementary file1 (DOCX 439 KB)

## Data Availability

The datasets generated during and/or analyzed during the current study are available from the corresponding author on reasonable request.
