# Supplementary material for: Development and external validation of a multivariable [68Ga]Ga-PSMA-11 PET-based prediction model for lymph node involvement in men with intermediate or high-risk prostate cancer
Source: Eur J Nucl Med Mol Imaging. 2023 Jun 1;50(10):3137–46. doi: 10.1007/s00259-023-06278-1 (PMC10382335; doi:10.1007/s00259-023-06278-1)
Supplement: Supplementary file 1 — Supplementary file1 (DOCX 439 KB) [file 259_2023_6278_MOESM1_ESM.docx]

**Supplementary Materials**

**Figures**


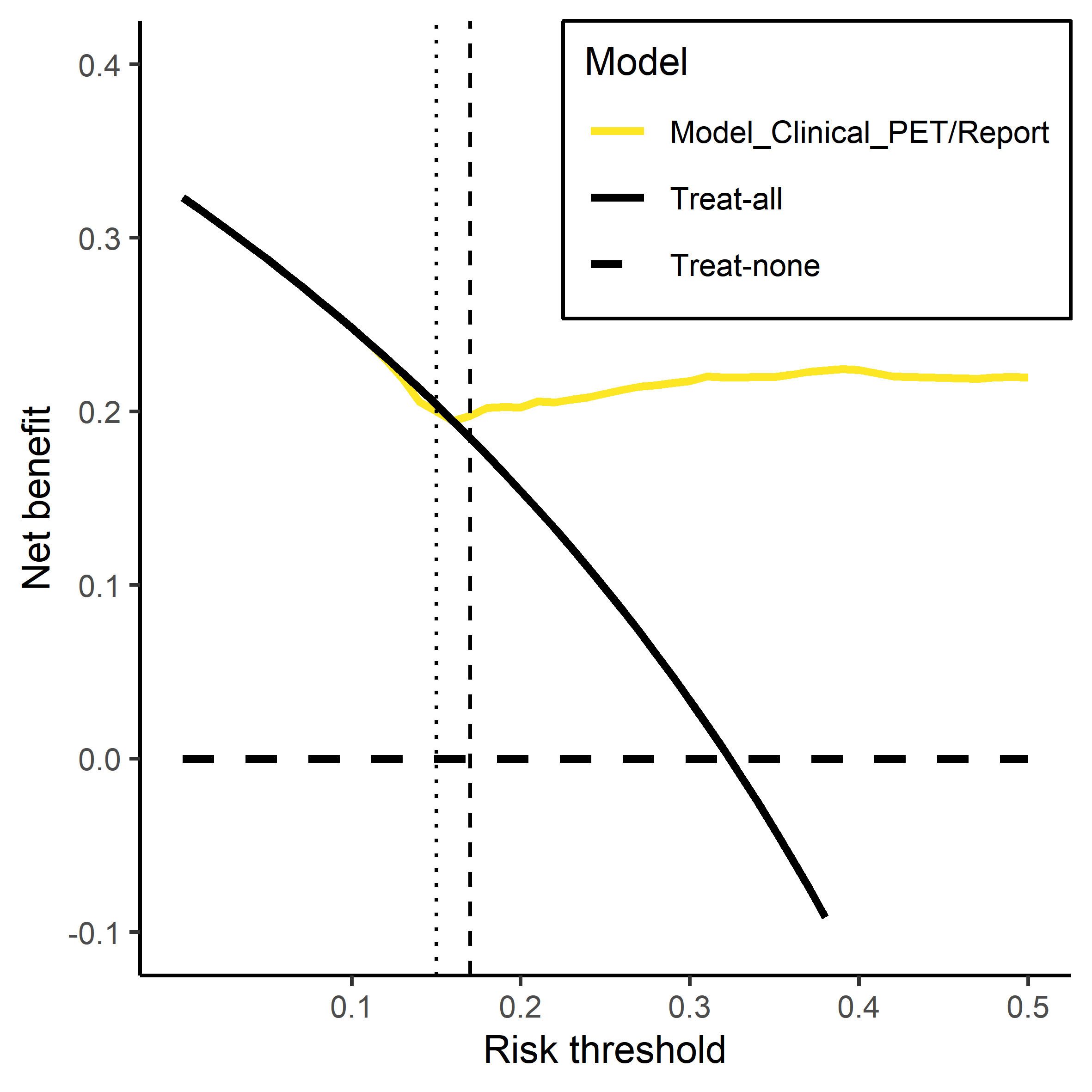


**Figure S1** - Cross-validated decision curve analysis of the ensemble model (Model_Clinical_PET/Report) in the training cohort. The vertical dotted line represents the risk threshold (15 %) for lymph node involvement where the model has a higher net benefit compared to a treat-all or treat-none strategy. The vertical dashed line represents the estimated optimal threshold (≥17 %) with an estimated spared ePLND of 54.3% and missed LNI of 19.1% that fitted best the reported corresponding values of the 5% threshold of the 2012 Briganti model (spared PLND of 65.5% and missed LNI of 12.2 %).


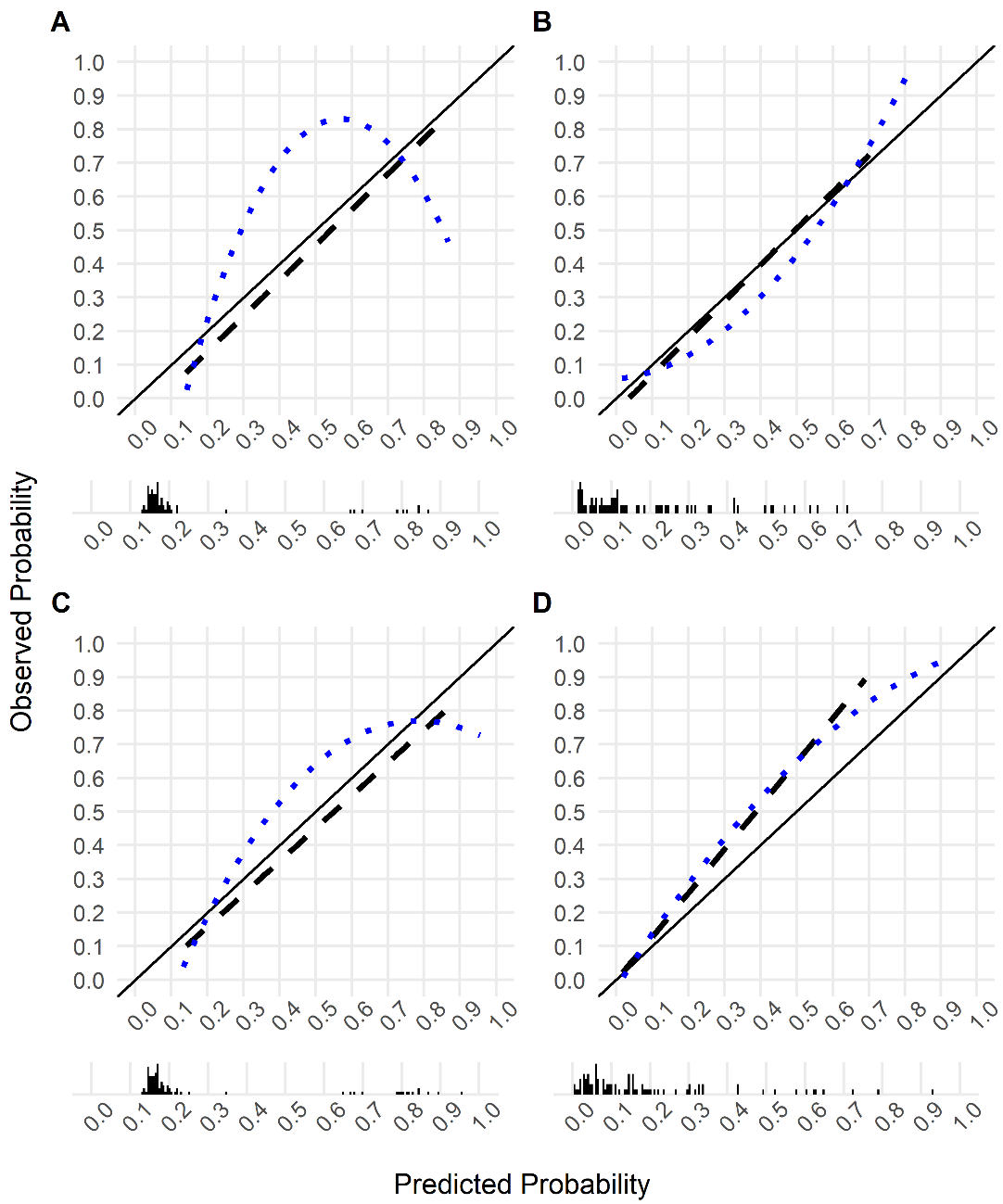


**Figure S2** - Model calibration plots of predicted probability versus observed probability of lymph node involvement for the complete case analyses. (A) Represents the proposed model (Model_Clinical_PET/Report) in the complete case analysis (n= 67) for (B) the 2019 Briganti model. (C) represents Model_Clinical_PET/Report in the complete case analysis (n = 86) of the (D) Draulans et. al model. The dotted lines represent the LOESS fit, the dashed lines represent a straight fit. The black bars denote the distribution of predicted probabilities.

**Tables**

**Table S1** - Additional data extraction for model comparison

| Model | Additional data extraction | Complete cases |
| --- | --- | --- |
| 2019 Briganti [1] | WHO/ISUP grade group at targeted biopsy  Percentage of cores with ISUP≥2 at systematic biopsy  T-Stage at mpMRI (organ-confined, extracapsular extension, seminal vesicle invasion)^a^  Maximum index lesion diameter at MpMRI (mm)^a^ | 67 |
| Draulans et. al. [2] | Maximum tumour length in 1 core (systematic and/or targeted biopsy)  WHO/ISUP grade group corresponding to maximum tumour length in 1 core  T-Stage on mpMRI (iT1c, iT2a-2b, iT2c, iT3a, iT3b, iT4)^a^ | 86 |
| MSKCC[22] | Clinical T-stage | 90 |

^a^Assessed in consensus and blinded regarding the pathological outcome by two radiologists on mpMRI that were acquired according to the PI-RADS guidelines.

**Table S2** ‑ Patient characteristics of the complete-case analyses for the 2019 Briganti and Draulans et al. prediction model.

| Model (n) | Characteristics | Validation cohort | | | |
| --- | --- | --- | --- | --- | --- |
|  |  |  | LNI- | LNI+ | P |
| 2019 Briganti (67,  LNI- 54, LNI+ 13)^a^ | PSA | Median | 8.4 | 11.7 | 0.23 |
|  |  | Range | 1.9 - 29.5 | 2.1 - 143 |  |
|  |  | IQR | 7.8 | 12.8 |  |
|  | WHO/ISUP grade group^b^ | 1 | 3 | 0 | 0.67 |
|  |  | 2 | 14 | 2 |  |
|  |  | 3 | 11 | 3 |  |
|  |  | 4 | 20 | 5 |  |
|  |  | 5 | 6 | 3 |  |
|  | Percentage of cores with ISUP≥2^c^ | Median | 13 | 33 | **0.003** |
|  |  | Range | 0 - 67 | 0 - 83 |  |
|  |  | IQR | 15 | 21 |  |
|  | T-Stage at mpMRI | organ-confined | 40 | 4 | **0.04** |
|  |  | extracapsular extension | 10 | 5 |  |
|  |  | seminal vesicle invasion | 4 | 4 |  |
|  | Maximum index lesion diameter at mpMRI (mm) | Mean | 14 | 25.5 | **0.004** |
|  |  | Range | 1.7 - 39 | 10 - 48 |  |
|  |  | SD | 7.4 | 11.4 |  |
| Draulans  (86, LNI- 65, LNI+ 21)^a^ | PSA | Median | 8.4 | 14 | **<0.001** |
|  |  | Range | 1.86 – 55 | 2.08 – 143 |  |
|  |  | IQR | 7.9 | 13.5 |  |
|  | Maximum tumour length in 1 core (mm)^d^ | Mean | 6.8 | 9.5 | **0.02** |
|  |  | Range | 1.6 -18 | 1 - 18 |  |
|  |  | SD | 3.9 | 4.5 |  |
|  | WHO/ISUP grade group^e^ | 1 | 5 | - | **0.04** |
|  |  | 2 | 22 | 5 |  |
|  |  | 3 | 20 | 3 |  |
|  |  | 4 | 13 | 8 |  |
|  |  | 5 | 5 | 5 |  |
|  | T-Stage on mpMRI | iT1c | 3 | - | **0.004** |
|  |  | iT2a-2b | 38 | 4 |  |
|  |  | iT2c | 9 | 3 |  |
|  |  | iT3a | 11 | 7 |  |
|  |  | iT3b | 3 | 6 |  |
|  |  | iT4 | 1 | 1 |  |

^a^ Eighteen patients of the validation cohort had missing data concerning the Briganti 2019 model (not adequate mpMRI: n=4, negative mpMRI: n=2, negative preoperative targeted biopsy: n=6, missing targeted biopsy results: n=5) and 4 patients for the Draulans et. al model (not adequate mpMRI: n=4), respectively, ^b^ At targeded biopsy,^c^ At systematic biopsy,^d^ At targeted and/or systematic biopsy, ^e^ Corresponding to maximum tumour length in 1 core

**Table S3** - Sensitivity/Specificity/PPN/NPV and AUC of nuclear medicine physicians reading PSMA PET in the training and evaluation cohort

| Measure | Threshold of PSMA-PET LNI report status of 0.5 (equivocal PSMA-PET considered positive) | Threshold of PSMA-PET LNI report status of 1 (equivocal PSMA-PET considered negative) |
| --- | --- | --- |
| Sensitivity  Training cohort  Evaluation cohort | 0.70 (95%-CI 0.56-0.81)  0.52 (95%-CI 0.30-0.74) | 0.54 (95%-CI 0.40-0.67)  0.43 (95%-CI 0.22-0.66) |
| Specificity  Training cohort  Evaluation cohort | 0.99 (95%-CI 0.95-1)  0.93 (95%-CI 0.84-0.98) | 0.99 (95%-CI 0.95-1)  0.97 (95%-CI 0.90-1) |
| Positive predictive value Training cohort  Evaluation cohort | 0.97 (95%-CI 0.87-1)  0.69 (95%-CI 0.41-0.89) | 0.97 (95%-CI 0.83-1)  0.82 (95%-CI 0.48-0.98) |
| Negative predictive value Training cohort  Evaluation cohort | 0.87 (95%-CI 0.80-92)  0.86 (95%-CI 0.77-0.93) | 0.82 (95%.CI 0.74-0.88)  0.85 (95%-CI 0.75-0.92) |
| Area under the curve  Training cohort  Evaluation cohort | 0.84 (95%-CI 0.78–0.91)  0.73 (95%-CI 0.61-0.84 | 0.76 (95%-CI 0.69-0.83)  0.30 (95%-CI 0.19-0.41) |

**Table S4 -** Model characteristics

| Model | Variable | Coefficient | SE | P | OR (95%-CI) |
| --- | --- | --- | --- | --- | --- |
| **Model_Clinical_PET** | (Intercept) | -2.379 | 0.583 | 0.000 | 0.09 (0.29-0.03) |
|  | ISUP | 0.222 | 0.145 | 0.126 | 1.25 (1.66-0.94) |
|  | PSA | 0.020 | 0.010 | 0.053 | 1.02 (1.04-1) |
|  | PSMA_vol_ | 0.051 | 0.016 | **0.001** | 1.05 (1.09-1.02) |
|  |  |  |  |  |  |
| **Model_Clinical_PET_Report** | (Intercept) | -2.302 | 0.788 | 0.003 | 0.1 (0.02-0.47) |
|  | PSMA PET report N-status | 6.734 | 1.475 | **0.000** | 840.34 (46.7-15122.64) |
|  | ISUP | 0.101 | 0.198 | 0.610 | 1.11 (0.75-1.63) |
|  | PSA | 0.004 | 0.018 | 0.831 | 1 (0.97-1.04) |
|  | PSMA_vol_ | 0.006 | 0.020 | 0.767 | 1.01 (0.97-1.05) |
|  |  |  |  |  |  |
| **Model_Clinical_PET/Report** |  |  |  |  |  |
| Model 1= Model_Clinical_PET | (Intercept) | -2.379 | 0.583 | 0.000 | 0.09 (0.03-0.29) |
|  | ISUP | 0.222 | 0.145 | 0.126 | 1.25 (0.94-1.66) |
|  | PSA | 0.020 | 0.010 | 0.053 | 1.02 (1-1.04) |
|  | PSMA_vol_ | 0.051 | 0.016 | **0.001** | 1.05 (1.02-1.09) |
|  |  |  |  |  |  |
| Model2 = Model_Report | (Intercept) | -1.868 | 0.254 | 0.000 | 0.15 (0.09-0.25) |
|  | PSMA PET report N-status | 6.840 | 1.425 | **0.000** | 934.85 (57.29-15254.2) |
| Weighted average function | Weight Model1 = 0.3232 | - | - | - | - |
|  | Weight Model2 = 0.9267 | - | - | - | - |

**Table S5** - Calibration characteristics of Model_Clinical_PET/Report compared to conventional prediction Models

| Model | Cohort n | Brier | Spiegelhalter’s z | Index of prediction accuracy |
| --- | --- | --- | --- | --- |
| Model_Clinical_PET/Report | 90 | 0.12 | -0.89 | 0.35 |
| MSKCC |  | 0.15 | 2.96 | 0.22 |
| Partin v.2016 |  | 0.19 | 5.65 | -0.12 |
| Roach |  | 0.16 | -1.18 | 0.17 |
| Winter |  | 0.17 | 0.29 | 0.12 |

**Table S6** - Calibration characteristics of Model_Clinical_PET/Report compared to multiparametric MRI based prediction models

| Model | Cohort n | Brier | Spiegelhalter’s z | Index of prediction accuracy |
| --- | --- | --- | --- | --- |
| Model_Clinical_PET/Report | 67 | 0.13 | -0.46 | 0.24 |
| 2019 Briganti |  | 0.11 | -0.60 | 0.37 |
| Model_Clinical_PET/Report | 86 | 0.13 | -0.71 | 0.34 |
| Draulans et al |  | 0.13 | 0.84 | 0.31 |

**References of the supplementary Material**

1. Gandaglia G, Ploussard G, Valerio M, et al (2019) A Novel Nomogram to Identify Candidates for Extended Pelvic Lymph Node Dissection Among Patients with Clinically Localized Prostate Cancer Diagnosed with Magnetic Resonance Imaging-targeted and Systematic Biopsies. European Urology 75:506–514. <https://doi.org/10.1016/j.eururo.2018.10.012>

2 Draulans C, Everaerts W, Isebaert S, et al (2020) Development and External Validation of a Multiparametric Magnetic Resonance Imaging and International Society of Urological Pathology Based Add-On Prediction Tool to Identify Prostate Cancer Candidates for Pelvic Lymph Node Dissection. Journal of Urology 203:713–718. <https://doi.org/10.1097/JU.0000000000000652>

3 MSKCC Pre-Radical Prostatectomy. <https://www.mskcc.org/nomograms/prostate/pre_op>
